# Total Arterial Revascularization in Diabetic Patients Undergoing Coronary Artery Bypass Graft Surgery: A Systematic Review and Meta-Analysis

**DOI:** 10.31083/j.rcm2406183

**Published:** 2023-06-25

**Authors:** Guang-zhi Liao, Ting Liu, Yi-ming Li, Lin Bai, Yu-yang Ye, Xue-feng Chen, Yong Peng

**Affiliations:** ^1^Department of Cardiology, West China Hospital, Sichuan University, 610041 Chengdu, Sichuan, China

**Keywords:** systematic review, meta-analysis, total arterial revascularization, diabetes, survival analysis

## Abstract

**Background:**

Total arterial revascularization (TAR) has gradually become accepted and recognized, but its effect and safety in diabetic patients are not clear. We performed a systematic review and meta-analysis to summarize the safety and efficacy of TAR and additionally evaluated the clinical outcomes of arterial revascularization using different arterial deployments in patients with diabetes.

**Methods:**

PubMed, Embase, and the Cochrane Library databases from inception to July 2022 for studies that studied the effect of arterial revascularization in diabetic patients undergoing isolated coronary artery bypass graft (CABG) were searched. The primary outcome was long-term (≥12 months of follow-up) death by any cause. The secondary efficacy endpoints were long-term (≥12 months) cardiovascular death, early sternal wound infection (SWI) and death (≤30 days or in hospital). Risk ratios (RRs), hazard ratios (HRs), and their corresponding 95% confidence intervals (CIs) were calculated to describe short-term results and long-term survival outcomes. Two different ways were used to analyze the effect of TAR and the impact of diabetes on the clinical outcomes of TAR.

**Results:**

Thirty-five studies were included in the study, covering 178,274 diabetic patients. Compared to conventional surgery with saphenous veins, TAR was not associated with increased early mortality (RR 0.77, 95% CI 0.48–1.23) and risk of SWI (RR 0.77, 95% CI 0.46–1.28). The overall Kaplan–Meier survival curves based on reconstructed patient data indicated a significant association between TAR and reduced late mortality (HR 0.52, 95% CI 0.48–0.67) and the curves based on the propensity-score matched (PSM) analyses suggested a similar result (HR 0.74, 95% CI 0.66–0.85). TAR could also effectively decrease the risk of cardiovascular death (HR 0.42, 95% CI 0.24–0.75). Through comparing the effect of TAR in patients with and without diabetes, we found that the presence of diabetes did not elevate the risk of early adverse events (death: RR 1.50, 95% CI 0.64–3.49; SWI: RR 2.52, 95% CI 0.91–7.00). Although diabetes increased long-term mortality (HR 1.06; 95% CI 1.35–2.03), the cardiovascular death rate was similar in patients with diabetes and patients without diabetes (HR 1.09; 95% CI 0.49–2.45). Regarding the selection of arterial conduits, grafting via the bilateral internal mammary artery (BIMA) decreased the risk of overall death (HR 0.67, 95% CI 0.52–0.85) and cardiovascular death (HR 0.55, 95% CI 0.35–0.87) without resulting in a significantly elevated rate of early death (RR 0.95, 95% CI 0.82–1.11). However, the evidence from PSM studies indicated no difference between the long-term mortality of the BIMA group and that of the single internal mammary arteries (SIMA) groups (HR 0.76, 95% CI 0.52–1.11), and the risk of SWI was significantly increased by BIMA in diabetes (RR 1.65, 95% CI 1.42–1.91). The sub-analysis indicated the consistent benefit of the radial artery (RA) application in diabetic patients (HR 0.71, 95% CI 0.63–0.79) compared to saphenous vein graft. In two propensity-score-matched studies, the evidence showed that the survival outcomes of the BIMA group were similar to that of the SIMA plus RA group but that grafting via the RA reduced the risk of sternal wound infection.

**Conclusions:**

Compared with conventional surgery using SVG, TAR was associated with an enhanced survival benefit in diabetes and this long-term gain did not increase the risk of early mortality or SWI. Given the increased infection risk and controversial long-term survival gains of grafting via the BIMA in diabetes, its wide use for grafting in this cohort should be seriously considered. Compared to using the right internal mammary artery (RIMA), RA might be a similarly effective but safer option for patients with diabetes.

## 1. Introduction

Coronary artery bypass grafting (CABG) has been identified as the preferred revascularization strategy for patients with multivessel disease and diabetes. As an important factor influencing the clinical outcomes of those receiving surgery, graft selection has gradually attracted investigators’ attention in recent years. Compared with conventional surgery involving saphenous venous grafts (SVGs), using the left internal mammary artery (LIMA) to bypass a stenotic left anterior descending artery (LAD) improves outcomes and is thus considered the standard of care. However, with SVGs failure rating up to 10% to 20% after 1 year and an additional 5% failure rate for each subsequent year [1, 2, 3], debates began to surround the application of additional arterial grafts. An increasing number of studies have detailed the association between total arterial revascularization (TAR) and improved long-term survival in the general population [4, 5, 6]. Nevertheless, before TAR can be widely performed in clinical practice, it needs further development because of its association with increased surgical difficulty and risks caused by some specific comorbidities, such as diabetes.

Oftentimes, patients with diabetes mellitus (DM) have complex, three-vessel coronary artery lesions. Consequently, surgeons usually have to carefully select the best graft to serve as the adjunct to the LIMA. Despite a prolonged operation time and increased surgical difficulty, the primary reasons hindering the application of multiarterial/ total-arterial coronary revascularization (MAR/TAR) are the increased risks of sternal wound infection and perioperative mortality. Therefore, whether DM patients can get consistent long-term benefits from arterial grafts, which may overweigh the short-term risk, is a critical issue that requires investigation. Moreover, the clinical outcomes of arterial revascularization via different arteries are also not clear. In this context, we conducted this systematic review and meta-analysis to provide the latest evidence to answer these issues above.

## 2. Methods

This study was registered on INPLASY (INPLASY2022120003). We performed and reported this work in accordance with the Preferred Reporting Items for Systematic Reviews and Meta-Analysis statements [7]. All data used in this study were extracted from individual studies. The authors declare that all supporting data are available within the article and the supplementary documents.

### 2.1 Literature Search

We searched PubMed, Embase, and Cochrane from inception to July 2022 for studies evaluating the outcome of arterial revascularization in diabetic patients undergoing isolated CABG. The search strategies and related terms are provided in the **Supplemental file**. Two reviewers (GL and TL) screened each study by title and abstract for inclusion eligibility, reviewed the full texts of eligible studies, and then extracted the data independently. All disagreements were resolved by discussion. The references of selected articles and conference proceedings were also screened.

### 2.2 Eligible Study and Endpoints of Interest

Inclusion criteria: (1) studies evaluating patients with a primary diagnosis of diabetes according to the International Classification of Diseases, 10th Revision, and receiving insulin or oral treatment before isolated CABG; (2) studies reporting on any one of the following comparisons: outcomes of TAR and conventional revascularization with veins (CVR) in diabetic patients, outcomes in diabetic and nondiabetic patients following TAR grafting, outcomes of BIMA/RA access and right gastroepiploic artery (RGA) access in patients with DM; (3) postoperative sternal wound infection rate (superficial and deep infections, SWIs), and Kaplan‒Meier survival curves of all-cause death and cardiovascular death or hazard ratio (HR) for the two outcomes; and (4) randomized and nonrandomized controlled trials published in English. We defined long-term (≥12 months) all-cause death as the primary endpoint of interest. The secondary efficacy endpoints were long-term (≥12 months) cardiovascular death, early SWI and death (occurred in hospital or within 30 days after surgery). We compared the outcome of TAR in patients with DM with that in patients without DM to investigate the influence of DM on the effect of arterial revascularization. To avoid minor study effects, studies with a sample size of <100 patients were excluded.

### 2.3 Data Extraction and Quality Assessment

Two researchers (LG and LT) independently extracted the following information from each work: the first author, publication year, type of study, and participant characteristics. The reviewers extracted the following outcomes of interest: early death, any SWI, any Kaplan–Meier curve for long-term overall survival, or cardiac mortality-free survival. For studies that reported the results of propensity-score–matched (PSM) analyses, we also abstracted and pooled the PSM data separately. When studies performed stratified analysis according to the number of arterial conduits used, we included the patients who received ≥3 arterial grafts as the TAR group and the patients who received only 1 arterial conduit therapy as the CVR cohort. The Cochrane risk of bias tool was used to examine randomized control trials (RCTs) [8], and the Newcastle-Ottawa Scale (NOS, http://www.ohri.ca/programs/clinical_epidemiology/nosgen.pdf) was used to investigate observational studies. Patients with a NOS score of less than 6 will be excluded. Sensitivity analysis was conducted to test the robustness of results, and publication bias was evaluated by visual inspection of funnel plots when the number of studies was greater than 10.

### 2.4 Statistical Analysis

Risk ratios (RRs), hazard ratios (HRs), and their corresponding 95% confidence intervals (CIs) were calculated to describe short-term results and long-term survival results. The $I^{2}$ statistics were performed to test for heterogeneity between the included studies, and a fixed-effects model was used to obtain the combined RRs and HRs when the $I^{2}$ statistic was lower than 50%. Otherwise, the random-effects model was alternatively adopted. Forest plots for outcomes of interest and sensitivity analyses were created with the package Meta of R software (R Foundation for Statistical Computing, Vienna, Austria. URL http://www.R-project.org/. version 4.1.3). Funnel plots were made for the comparisons with 10 or more studies included. In each included study, Engauge Digitizer version 11.1 (free software downloaded from http://sourceforge.net) was used to extract the time and the survival rate at the corresponding time point from the survival curve. The HR calculations spreadsheet [9] [https://static-content.springer.com/esm/art%3A10.1186%2F1745-6215-8-16/MediaObjects/13063_2006_188_MOESM1_ESM.xls] was then applied to facilitate the estimation of HRs from the data extracted by Engauge Digitizer. We applied the Meta and Forest plot packages of R software (version 4.1.3) to pool HRs and generate the corresponding forest plots. Additionally, to construct combined survival curves, Kaplan–Meier (KM) survival curves for long-term death-free survival and digitalized KM curve data were aggregated using the package MetaSurv of R software (URL http://www.R-project.org/. version 3.4.3) [10].

## 3. Results

### 3.1 Study Characteristics and Quality

**Fig. 1.**
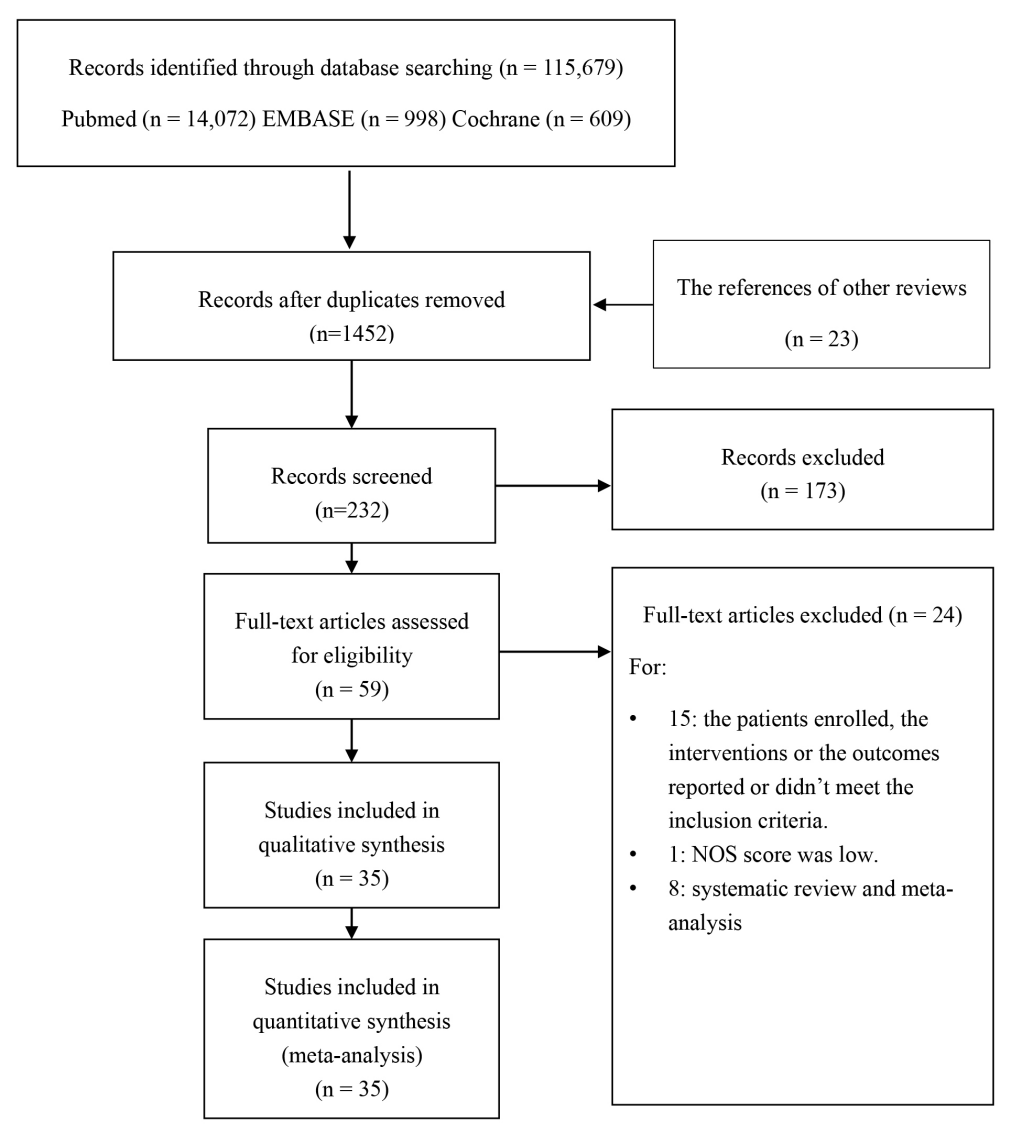
**The flow diagram of literature searching and selection**.

Fig. 1 details the PRISMA systematic review flowchart. After review, a total of 34 observational studies and one RCT [4, 11, 12, 13, 14, 15, 16, 17, 18, 19, 20, 21, 22, 23, 24, 25, 26, 27, 28, 29, 30, 31, 32, 33, 34, 35, 36, 37, 38, 39, 40, 41, 42, 43, 44], covering 178,274 diabetic patients, were included in this meta-analysis. The characteristics of the included studies are provided in Table 1 (Ref. [4, 11, 12, 13, 14, 15, 16, 17, 18, 19, 20, 21, 22, 23, 24, 25, 26, 27, 28, 29, 30, 31, 32, 33, 34, 35, 36, 37, 38, 39, 40, 41, 42, 43, 44]). The average duration of follow-up in the studies evaluating the long-term outcomes was 71.1 months. In all the studies, the mean age was 63.8 years, with an apparent male predominance (75.1%). Obesity, chronic cardiac insufficiency, chronic renal disease and pulmonary insufficiency were common in the setting of diabetes. Among the studies exploring the effect of TAR [4, 14, 16, 17, 22, 38, 41]. RA was the artery most frequently selected as the adjunct to the LIMA. After reviewing these studies, the overall patient profile was similar between the groups. The funnel plots of the comparisons of the BIMA and the SIMA suggested the possible existence of publication bias. The overall risk of bias was considered moderate in the RCT. Except for the study of Raza *et al*. [34] published in 2013, the quality evaluation of non-RCTs based on the Newcastle-Ottawa scale found that all scores were ≥6 (**Supplementary Table 1** in the **Supplemental file**).

**Table 1.** **Baseline characteristics of individual studies**.

Study	Pts	Study design	Trial group	The major arterial conduct	Control group	Study period	Follow-up, m	Age, y	Male (%)	Prior MI (%)	Prior Re. (%)	Obesity, %/BMI, kg/$m^{2}$	HBA1c (%) (mean)	Diabetes on insulin (%)	CHI/LVEF (%)	CKD (%)	Pulmonary insufficiency/COPD (%)	Smoker (%)	NOS or Rob2
Buxton 2012 [14]	206	Retrospective; PSM	TAR in DM	RA	CVR in DM	1996–2008	93.6	Age >70 years: 35.9	84.0	50.5	10.2	18.9 /NA	NA	18.9	1.9	Creatinine >200: 2.4	3.9	23.3	8
DiBacco 2019 [17]	269	Retrospective; PSM	TAR in DM	BIMA and RA	CVR in DM	2005–2015	101	70.1	77.3	40.4	16.7	NA	NA	13.6	NA/50.2	12.1	13.1	NA	9
Tatoulis 2015 [4]	11,642	Retrospective; PSM	TAR in DM	RA	CVR in DM	2001–2012	58.8	66.0	75.7	56.2	14.6	NA/30.0	NA	NA	19.1/NA	Preoperative dialysis: 2.7	12.7	66.1	8
Hwang 2010 [22]	558	Retrospective	TAR in DM	BIMA and RGA	TAR in NDM	1998–2004	81	61.7	75.1	NA	NA	42.7/NA	NA	13.6	6.1 (LVEF <0.35)/NA	5.9	NA	45.3	8
Suzuki 2015 [38]	602	Retrospective	TAR in DM	BIMA and RGA	TAR in NDM	2002–2013	52.8	67.0	84.1	33.1	29.4	NA/23.8	HbA1c ≥6.1%	29.6	27.4	10.1	17.9	63.3	9
Choi 2005 [16]	517	Prospective	TAR in DM	BIMA and RGA	TAR in NDM	1998–2003	34.0	61.4	76.0	19.9	NA	1.5/NA	NA	12.6	6.0	5.6	NA	45.1	7
Schwann 2018 [41]	3992	Retrospective; PSM	TAR in DM	RA	TAR in NDM; CVR in DM	1994–2011	104.4	64.0	66.8	54.4	19.0	NA/>25: 80.9	NA	NA	15.2/48.0	0	22.2	NA	9
Muneretto 2006 [31]	200	Retrospective; PSM	BIMA	BIMA	SIMA	1999–2003	34	68.5	58.0	56.0	NA	11.0/NA	NA	NA	NA/LVEF <30%: 11.0	NA	26.0	13.0	7
Lev-Ran 2004 [28]	285	Retrospective	BIMA	BIMA	SIMA	1996–1998	63.0	65.8	66.9	Acute MI <1 week: 20.0	14.0	NA/25.7	NA	0	21.0/NA	NA	NA	NA	8
Lev-Ran 2003 [29]	124	Retrospective	BIMA	BIMA	SIMA	1996–2001	55.0	65.9	58.9	NA	28.2	NA/25.0	NA	100.0	37.9/NA	13.7	11.3	37.1	8
Abelaira 2021 [12]	152	Retrospective	BIMA in diabetics	BIMA	BIMA in non-diabetics	2004–2017	3.0	62.2	85.0	34.2	NA	23.0/NA	5.9	78.7	15.8 (LVEF% < 40%)/NA	Hemodialysis: 1.3	14.5	42.1	7
Agrifoglio 2008 [13]	81	Prospective; PSMPU	BIMA	BIMA	SIMA	2006	12.0	66.5	64.4	34.1	NA	NA/27.4	8.4	34.6	NA/55.9	9.9	12.3	64.2	7
Dorman 2012 [18]	828	Retrospective: PSM	BIMA	BIMA	SIMA	1972–1994	106.8	65.7	79.3	57.7	NA	NA	NA	NA	15.0/LVEF <50%: 36.6	1.0	NA	58.2	8
Calafiore 2004 [15]	1140	Prospective: PSM	BIMA	BIMA	SIMA	1986–1999	87.6	60.8	81.6	49.0	0.0	NA /NA	NA	NA	2.8/59.4	2.3	2.9	NA	9
Endo 2003 [19]	467	Retrospective; PSM	BIMA	BIMA	SIMA	1985–1998	97.2	61.6	80.1	67.2	NA	60.0/NA	NA	10.7	NA/52.2	NA	NA	71.3	8
Gansera 2017 [20]	250	Retrospective: PSM	BIMA	BIMA	SIMA	2000–2011	111.6	59.7	83.2	35.1	20.0	NA	NA	38.0	34.8 (LVEF% < 50%)/NA	NA	NA	NA	7
Hirotani 2003 [21]	303	Retrospective	BIMA	BIMA	SIMA	1991–2003	NA	64.4	75.9	80.2	3.0	NA	NA	49.2	48.4/NA	NA	NA	NA	7
Iribarne 2017 [23]	430	Retrospective: PSM	BIMA	BIMA	SIMA	1992–2014	111.6	NA	73.9	MI within 7 days 14.9	17.4	NA/NA	11.7	NA	15.1/NA	5.9	11.7	NA	8
Kainuma 2021 [24]	124	Retrospective	BIMA	BIMA	SIMA	1995–2015	68.0	68.0	87.1	NA	NA	NA/23.0	7.1	40.3	100.0/32.8	eGFR <30 mL/min/1.73 $m^{2}$: 14.5	NA	NA	8
Kazui 2021 [25]	16,741	Retrospective: PSM	BIMA	BIMA	SIMA	2008–2016	1.0	60.0	84.5	NA	NA	NA/29.7	NA	NA	13.1/53.6	Dialysis 1.6	17.8	32.4	8
Konstanty-Kalandyk 2012 [27]	147	Retrospective	BIMA	BIMA	SIMA	2006–2008	3.0	65.0	61.2	67.3	NA	BMI >30 (kg/$m^{2}$): 38.1/28.7	NA	52.4	NA/51.2	8.8	6.8	NA	6
Kinoshita 2010 [26]	340	Retrospective: PSM	BIMA	BIMA	SIMA	2002–2009	38.4	69.5	75.3	45.0	28.5	NA/23.1	6.2	48.8	23.5 (LVEF <40%)/54.5	27.6	19.4	50.3	8
Momin 2005 [30]	920	Retrospective	BIMA	BIMA	SIMA	1992–2002	120.0	63.4	71.0	59.3	NA	NA/28.3	NA	28.4	NA/LVEF <50%: 48.6	17.4	6.2	11.5 (current); 57.2 (history of smoking)	7
Pevni 2017 [32]	980	Retrospective: PSM	BIMA	BIMA	SIMA	1996–2010	146.4	Age >70 years: 44.8	70.7	Recent MI <3 months: 33.0	23.4	NA/ BMI ≥30 kg/$m^{2}$: 12.2 (unmatched)	NA	14.8	28.8/NA	15.8	7.3	NA	8
Puskas 2012 [33]	1445	Retrospective	BIMA	BIMA	SIMA	2002–2010	108	62.6	70.9	53.0	NA	NA/29.4	NA	NA	21.2/50.2	6.8	14.7	60.3	8
Raza 2017 [11]	564	Retrospective; PSM	BIMA	BIMA	SIMA+RA	1994–2011	88.8	58.0	88.1	52.1	NA	NA/29.0	NA	NA	Left ventricular dysfunction: 44.3/NA	NA	5.5	NA	8
Raza 2014 [34]	9404	Retrospective	BIMA	BIMA	SIMA	1972–2011	93.6	62.0	72.1	56.5	NA	NA/30.0	NA	23.0	15.7/NA	2.4 (dialysis)	NA	NA	8
Sajja 2012 [35]	1211	Retrospective	BIMA	BIMA	SIMA	2004–2010	During the hospitalization.	58.2	86.8	28.7	NA	NA/25.9	NA	NA	LVEF <40%: 10.1/ NA	Serum creatinine >1.3 mg: 39.0	15.9	23.0	6
Savage 2006 [36]	120,793	Retrospective	BIMA	BIMA	SIMA	2002–2004	<30 days	64.6	67.7	46.2	NA	NA/30.8	NA	29.6	19.6/NA	8.8	19.6	19.1	6
Stevens 2005 [37]	633	Retrospective	BIMA	BIMA	SIMA	1985–1995	132	62.0	72.0	30.3	0.6	24.1/NA	NA	NA	1.6	NA	5.9	NA	9
Taggart 2019 [39]	734	RCT	BIMA	BIMA	SIMA	2004–2007	Last 120 months	63.6	85.6	41.9	15.8	NA/28.2	NA	23.7	NA/NA	NA	NA	70.4	Some concerns
Tavolacci 2003 [40]	256	Retrospective	BIMA	BIMA	SIMA	1998–2000	NA	66.2	78.3	NA	NA	NA	NA	NA	NA/NA	NA	NA	NA	7
Toumpoulis 2006 [42]	980	Retrospective; PSM	BIMA	BIMA	SIMA	1992–2002	56.4	64.1	55.6	56.9	11.4	NA/BMI ≥24: 18.4%	NA	NA	21.5/LVEF <30%: 19.9%	3.6	16.4	15.6	8
Hoffman 2014 [44]	404	Retrospective: PSM	RA	RA	BIMA	1995–2012	126.8	61.9	66.3	NA	17.8	NA	NA	NA	45.2	15.6	11.4	NA	8
Puehler 2020 [43]		Retrospective: PSM	BIMA	BIMA	SIMA	2009–2016	36.3	59.8	88.4	27.2	Previous surgery 1.2	NA/28.3	NA	NA	NA/58.0	NA	4.6	51.9	8

Pts, patients; m, months; y, years; MI, myocardial infarction; Re, revascularization; HBA1c, glycosylated hemoglobin, type A1C; CHI, chronic cardiac insufficiency; EF, ejection fraction; CKD, chronic kidney disease; COPD, chronic obstructive pulmonary disease; PSM, propensity score matching; TAR, total arterial revascularization; SIMA, single internal mammary arteries; BIMA, bilateral internal mammary arteries; RA, radial artery; RGA, right gastroepiploic artery; DM, diabetes mellitus; NDM, non-diabetes Mellitus; NA, not available; BMI, body mass index; eGFR, estimated glomerular filtration rate; CVR, conventional revascularization with veins.

### 3.2 The Early and Late Outcomes of TAR and CVR in DM

**Fig. 2.**
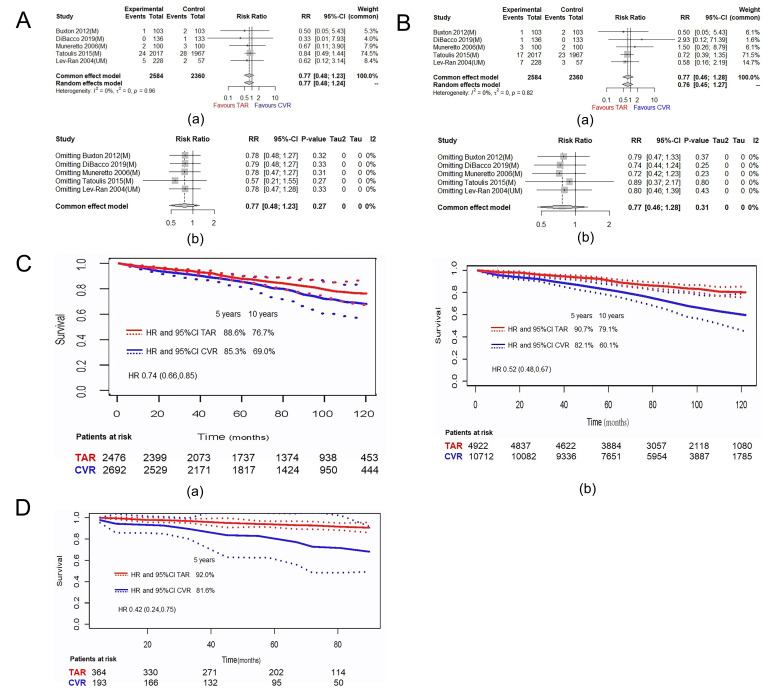
**Plots for the clinical outcomes of TAR in diabetic patients**. (A) (a) Forest plot of early death; (b) Forest plot of sensitivity analysis. (B) (a) Forest plot of any SWI; (b) Forest plot of sensitivity analysis. (C) Overall Kaplan–Meier survival curves based on reconstructed patient data. (a) Aggregated survival curve for long-term overall survival with data of 4 propensity score matched analyses; (b) Aggregated survival curve for long-term overall survival with data of all the cohorts from 5 studies. (D) Kaplan-Meier curves in the diabetic population for survival free from cardiovascular death based on reconstructed patient data from 1 propensity score matched analyses and 1 research with unmatched cohorts. Note: M: the studies with data of matched cohorts; UM: the studies with data of unmatched cohorts; TAR, total arterial revascularization; CVR, conventional revascularization with veins; HR, hazard ratio; CI, confidence interval.

Five observational studies enrolling 15,634 eligible patients, with NOS scores ranging from 7 to 9, were included in the analysis [4, 14, 17, 28, 31, 41]. The PSM method was not utilized in one study [28]. In the study by Lev-Ran, *et al*. [28], as TAR was performed in 91% of the patients in the BIMA group, we included this article in this analysis. The incidence of early death and any SWI in patients with DM are shown in Fig. 2A,B. Compared to conventional surgery with SVG, TAR was not associated with an increased risk of early mortality (RR 0.77, 95% CI [0.48–1.23]) or risk of SWI (RR 0.77, 95% CI [0.46–1.28]). The results of the sensitivity analyses suggest that no study contributes to residual heterogeneity. Removing them from the meta-analysis one by one would not influence the results. None of the $I^{2}$ value (0%) suggested significant heterogeneity. According to the aggregated survival curve for long-term overall survival with data from PSM analyses [4, 14, 17, 41], TAR improves long-term overall survival in DM (HR 0.74, 95% CI [0.66–0.85]). The 5-year and 10-year survival rate in the TAR and CVR arms were respectively 88.6%, 76.7% and 85.3%, 69.0% (Fig. 2C(a)). The survival curve of all patients from 5 studies suggested a consistent finding (HR 0.52, 95% CI [0.48–0.67]) (Fig. 2C(b)) [4, 14, 17, 28, 41]. Only the data from two studies were available for cardiovascular death analysis [17, 28]. Similarly, the patients who underwent TAR had a higher survival free from cardiovascular death (Fig. 2D) (HR 0.42, 95% CI [0.24–0.75]), at the 90-month follow-up.

### 3.3 The Early and Late Outcomes of TAR in DM and non-DM

Four observational studies with 1677 patients provided information related to the outcome of TAR in DM and non-DM patients [16, 22, 38, 41]. Three of them did not utilize PSM method to conduct analysis. In the comparisons, as **Supplementary Fig. 1** (**Supplemental file**) shows, the risk of early death (RR 1.50, 95% CI 0.64–3.49) and infection (RR 2.52, 95% CI 0.91–7.00) did not differ between the DM and non-DM groups (**Supplementary Fig. 1A**). The long-term overall survival rate of diabetic patients was lower than that of patients without diabetes (HR 1.66; 95% CI 1.35–2.03) while cardiovascular survival rate was similar (HR 0.98; 95% CI 0.51–1.90) (**Supplementary Fig. 1B**).

### 3.4 Arterial Revascularization with BIMA

**Fig. 3.**
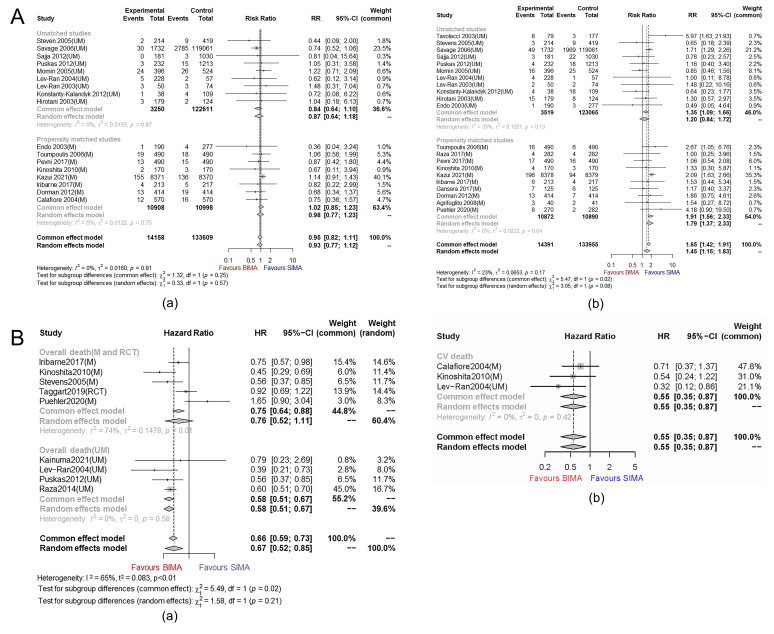
**Plots for the clinical outcomes of BIMA and SIMA in diabetic patients**. (A) (a) Forest plot of early death in matched and unmatched diabetic cohorts; (b) Forest plot of any SWI in matched and unmatched diabetic cohorts. (B) (a) Forest plot of long-term death among matched and unmatched cohort (85.7 months of average follow-up duration); (b) Forest plot of cardiovascular death among matched and unmatched diabetic cohorts (63 months of average follow-up duration). Note: M: the studies with data of matched cohorts; UM: the studies with data of unmatched cohorts; TAR, total arterial revascularization; CVR, conventional revascularization with veins; HR, hazard ratio; CI, confidence interval; DM, diabetes mellitus patients; non-DM, non-diabetes mellitus patients.

Twenty-three [13, 15, 18, 19, 20, 21, 23, 24, 25, 26, 27, 28, 29, 30, 32, 33, 35, 36, 37, 39, 40, 42, 43] studies with NOS scores ranging from 6 to 9 reported the early outcomes of the BIMA and the SIMA as adjuncts in DM. The short-term risk and long-term effects of BIMA as an access point are shown in Fig. 3. Compared to the SIMA, the BIMA was associated with a decreased risk of all-cause death (HR 0.67, 95% CI 0.52–0.85) and CV death (HR 0.55, 95% CI 0.35–0.87) without resulting in a significantly increased rate of early death (RR 0.95, 95% CI 0.82–1.11) (Fig. 3B,A(a)). The results of PSM studies (RR 0.84, 95% CI 0.64–1.10) and non-PSM researches in the analysis of early death were consistent (RR 1.02, 95% CI 0.85–1.23). However, the pooled analysis of 4 PSM studies and one RCT suggested no significant difference in survival gains (HR 0.76, 95% CI 0.52–1.11) between BIMA and SIMA in diabetes (the result of the random effects model was adopted as $I^{2}$ was 74%) (Fig. 3B(a)). Besides, the selection of the right internal mammary artery significantly increased the occurrence of SWI in DM (RR 1.65, 95% CI 1.42–1.91) (Fig. 3A(b)). Unmatched (RR 1.35, 95% CI 1.09–1.66) and PSM studies (RR 1.91, 95% CI 1.56–2.33) provided similar information. The sensitivity analyses (**Supplementary Fig. 2**) indicated the results robustness.

### 3.5 Arterial Revascularization with the RA and the RGA

**Fig. 4.**
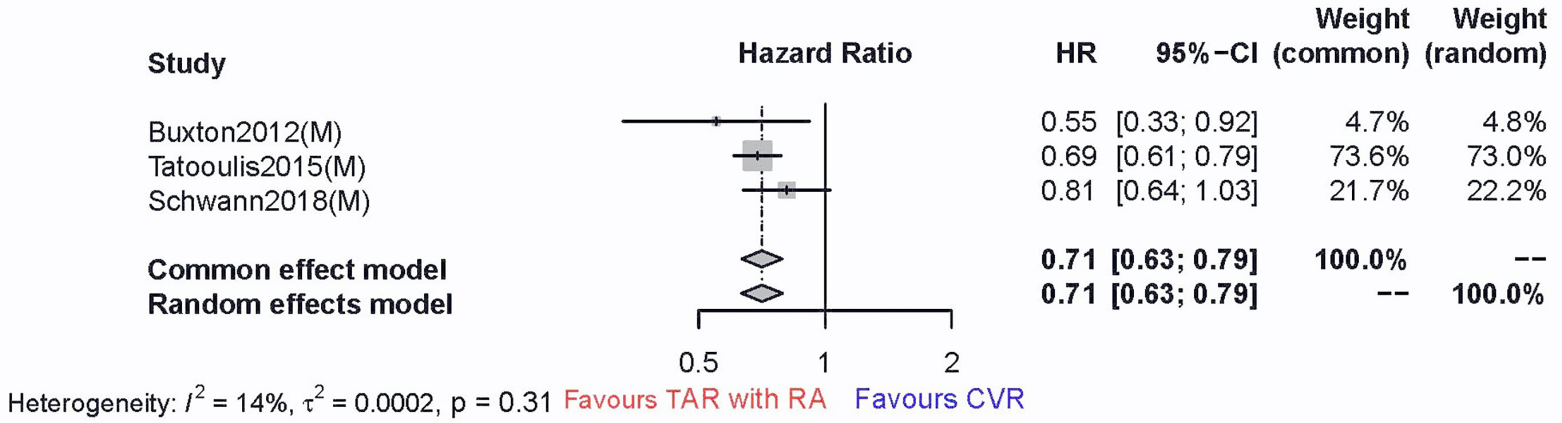
**The comparison between the all-cause mortality of TAR with RA versus that of CVR in diabetic patients (85.6 months of average follow-up duration)**. TAR, total arterial revascularization.

The available literature on the value of the RA and the RGA was limited. As Table 1 shows, most diabetic patients receiving TAR were treated via the RA as the arterial conduit second to the LIMA. the method of LIMA plus RA was applied in more than 80% of participants in the studies by Buxton, Tatoulis, and Schwan [4, 14, 41]. Therefore, we conducted an additional subanalysis for the primary outcome. The results suggested a consistent long-term survival benefit (HR 0.71, 95% CI 0.63–0.79) (Fig. 4). The sensitivity analyses (**Supplementary Fig. 3**) indicated the results robustness. After reviewing these works, we observed no differences in the risks of early death and SWI between the TAR group in which the RA was used and the CVR group. We failed to conduct additional analysis of RGA due to the absence of research.

### 3.6 The Comparisons between RIMA and RA

Only two PSM analyses, with a NOS score of 8, effectively compared the RIMA with the RA in patients with DM [11, 44]. In the propensity-matched analyses by Raza, *et al*. [11], in-hospital mortality risk (0.35% versus 0.35%), the prevalence of deep SWI (1.4% versus 1.4%) and overall survival rate were similar (*p* = 0.2) in the LIMA plus RA and BIMA groups. Supporting this finding, Hoffman’s PSM analysis indicated that long-term mortality was not significantly different between the use of RA and RIMA (*p* = 0.01) [44]. However, deep sternal wound infection (*p* < 0.035) favored the RA group.

## 4. Discussion 

Our work demonstrated that compared to conventional surgical revascularization with LIMA plus SVG, TAR was associated with a higher rate of long-term overall survival in diabetic patients, without being associated with a significantly increased risk of mortality or SWI. The presence of diabetes did not increase the risk of early death, SWI and long-term cardiovascular death. Regarding the selection of adjunct arteries second to the LIMA, the additional use of RIMA and RA could both exert the consistent survival benefit but did not increase early death. However, the BIMA method was found to result in more occurrences of SWI. Compared to the RIMA, RA might be a similarly effective but safer selection when TAR was applied in DM.

In recent years, multi-arterial grafting has been proven to improve survival rate in general population and is recommended by an increasing number of researchers [45, 46]. Gaudino *et al*. [45] extensively reviewed the benefits of arterial revascularization in a general population with multivessel disease. In their previous meta-analysis, the use of a third arterial conduit was not associated with a higher operative risk but was associated with superior long-term survival, irrespective of sex and diabetes [45]. However, the outcomes of arterial revascularization in diabetic patients were not well-defined and controversial, hampering its extended application in clinical practice. In this context, our work first systematically summarized the effect and safety of arterial grafts in this specific cohort. According to the results, this surgical approach did not increase perioperative death and SWI risk, but improve the survival rate significantly in diabetic patients. Therefore, the consideration on the perioperative events shouldn’t be the barrier excessively hindering the implementation of TAR into cardiac surgery. To explain the mechanisms underlying the survival gains, we found that the several excellent properties of arterial graft might contribute to this phenomenon. The thin smooth muscle layer and abundant elastic fibers of arterial conduits are relatively protected against the progression of atherosclerosis, resulting in better graft patency compared to SVG. In DM patients, the endothelial function of the coronary artery is depressed, resulting in a decrease in NO and prostacyclin secretion in the coronary artery circulation [47, 48]. In this context, arterial grafts transplanted into the coronary artery system can function not only as a nondiseased living conduit but also as a source of favourable metabolic substances that protect the coronary artery from atherosclerotic progression [49]. Theoretically, better graft patency and salutary metabolic effects on the recipient coronary arteries can lead to survival benefits, especially in DM patients with advanced atherosclerosis and depressed endothelial function.

It is well-known that using the LIMA to bypass a stenotic LAD artery is considered routine in patients eligible for surgery [50]. TAR/MAR has been advocated in recent years, so the selection of arterial conduits second to the LIMA has become a popular topic for discussion. The use of the RIMA was shown to be associated with enhanced survival benefits in people with or without diabetes in previous reviews and meta-analyses [51]. Although a higher occurrence of SWI was observed, they found that the incidence can be reduced by controlling perioperative blood glucose [52] and harvesting in a skeletonized fashion [10, 53]. Our work suggested similar overall survival gains and significantly increased SWI risk. Of note, however, the results of PSM studies and non-PSM analyses differ, and the considerable heterogeneity between researchers made this conclusion less reliable. The Inconsistencies in research indicated that the use of the BIMA in diabetic patients should be re-considered and treated seriously [39, 43]. On the one hand, significantly increased risks of SWI and prolonged preparation and operative times in DM patients require us to balance the pros and cons of accessing the BIMA. On the other hand, advances in medical treatment may narrow the differences in therapeutic effects between arterial revascularization and conventional surgery in the future. Currently, the crossover in the ART trial, one important RCT with a negative result, and multiple confounders in the observation studies made the survival gains of BIMA not very clear. Although most trials supported its application in DM patients, the debate over the selection of arterial grafts is ongoing, and the ROMA (Randomization of Single vs. Multiple Arterial Grafts, NCT03217006) trial, a large RCT in progress comparing the effect of TAR and CVR, is expected to provide more information and answers.

The RA is also often used as an adjunct to the LIMA and some RCTs have demonstrated its effectiveness in reducing the adverse outcomes in general population, compared with using SVG [54, 55]. However, studies exploring the outcomes of RA in DM are rather limited. The additional analysis of the three studies [4, 14, 41] applying RA as the second arterial grafting demonstrated the consistent long-term survival benefit. And they consistently reported no significant difference in the incidence of adverse early events when compared to the incidence of those associated with traditional surgery. Further systematic analysis of the comparisons between the RIMA and the RA was hampered by the limited number of relevant studies. According to the two PSM analyses, for diabetic patients, SIMA plus RA grafting and BIMA grafting yielded similar long-term survival after CABG. However, accessing the RA instead of RIMA can decrease the risk of SWI and thus might be the preferred conduit for more diabetic patients. While the RA also has its inherent flaws. For instance, it has been proved that RA is more prone to spasms in response to endogenous vasoconstrictors administered to DM patients [56]. Therefore, further data related to the effect comparisons of various arterial deployments from clinical trials are needed to improve the clinical outcomes of TAR in DM.

This study had several limitations. First, the majority of the studies discussed above were based on retrospective rather than prospective longitudinal data, reflecting outcomes after clinical decision-making by treating surgeons. Several studies were not evenly propensity-score matched, so there is a strong possibility of bias due to confounding. Especially in the comparisons between the outcomes of TAR in DM patients and that in non-DM patients, only one study used PSM method. Therefore, the results of analysis should be treated cautiously. Second, we could not study the freedom from clinical events such as recurrent MI, angina, cardiac death, or the need for repeat revascularization as these data were not available to us. Third, therapy using the BIMA grafts was different from applying TAR as other venous conduits can be used. Since studies were limited, we could not assess the outcomes of TAR using different arterial conduits. The outcomes of revascularization using the BIMA grafts was influenced by the application of venous conduits. Last, an evaluation of the RGA effect in DM was not performed due to the absence of relevant research and we failed to systematically summarize the corresponding effect.

## 5. Conclusions

Compared with conventional surgery using SVG, TAR was associated with an enhanced survival benefit in DM patients, but not the increased risk of early death and SWI. Given the increased infection risk and uncertain long-term survival gains of using the BIMA in DM patients, its wide use in this cohort should be seriously and cautiously considered. Compared to applying the RIMA, the RA might be a similarly effective but safer option for diabetic patients. However, the reliance of evidence was subjected to the limitation of observational studies and the findings above require the support of RCTs in the future.

## Data Availability

The datasets generated and analyzed during the current study are available from the corresponding author upon reasonable request.

## Abbreviations

CABG, coronary artery bypass graft; TAR, total arterial revascularization; MAR, multiple arterial revascularizations; CVR, conventional revascularization with venous grafts; DM, diabetes mellitus; BIMA, bilateral internal mammary arteries; LIMA, left internal mammary artery; RA, radial artery; RIMA, right internal mammary artery; RGA, right gastroepiploic artery; SVG, saphenous venous grafts; SWI, sternal wound infection; PSM study, propensity-score–matched study; CV death, cardiovascular death.

## Supplementary Material

Supplementary material associated with this article can be found, in the online version, at https://doi.org/10.31083/j.rcm2406183.
