# Supplementary material for: Total Arterial Revascularization in Diabetic Patients Undergoing Coronary Artery Bypass Graft Surgery: A Systematic Review and Meta-Analysis
Source: Rev Cardiovasc Med. 2023 Jun 25;24(6):183. doi: 10.31083/j.rcm2406183 (PMC11264116; doi:10.31083/j.rcm2406183)
Supplement: Supplementary file 1 [file 2153-8174-24-6-183-s1.zip › 2153-8174-24-6-183-s1/supplemental file.docx]

**Supplemental file**

**Supplement to:**

**Total Arterial Revascularization in Diabetic Patients Undergoing Coronary Artery Bypass Graft Surgery: A Systematic Review and Meta-analysis**

**Search strategy**

Pubmed (14072)

#1 diabetes mellitus[MeSH Terms] 492737

#2 ((diabetes[Title/Abstract]) AND (hyperglycemia[Title/Abstract])) AND (diabetes mellitus[Title/Abstract]) 13698

#3 "arterial coronary bypass"[Title/Abstract] OR "arterial revascularization"[Title/Abstract] OR "arterial conduit"[Title/Abstract] OR "mammary artery"[Title/Abstract] OR "internal thoracic artery"[Title/Abstract] OR "RIMA"[Title/Abstract] OR "BIMA"[Title/Abstract] OR "radial artery"[Title/Abstract] OR "gastroepiploic artery"[Title/Abstract] OR "RGA"[Title/Abstract] 19689

#4 ((coronary artery bypass graft[Title/Abstract]) OR (surgery[Title/Abstract])) OR (CABG[Title/Abstract]) 1,444284

#5 #1 OR #2 496828

#6 #3 OR #4 1457401

#7 #5 AND #6 14072

Cochrane library (609)

#1 MeSH descriptor: [Diabetes Mellitus] explode all trees 35999

#2 (diabetes):ti,ab,kw OR (diabetes mellitus):ti,ab,kw OR (hyperglycemia):ti,ab,k 99455

#3 #1 OR #2 102138

#4 (arterial coronary bypass):ti,ab,kw OR (arterial revascularization):ti,ab,kw OR (arterial conduit):ti,ab,kw OR (mammary artery):ti,ab,kw OR (internal thoracic artery):ti,ab,kw 3815

#5 (RIMA):ti,ab,kw OR (BIMA):ti,ab,kw OR (radial artery):ti,ab,kw OR (gastroepiploic artery):ti,ab,kw OR (RGA):ti,ab,kw 2768

#6 #4 OR #5 6334

#7 #3 AND #6 609

Embase (998)

#1 'diabetes mellitus':ta,ab OR diabetes:ab,ta OR 'hyperglycemia':ab,ta 943158

#2 'arterial coronary bypass':ta,ab OR 'arterial revascularization':ta,ab OR 'arterial conduit':ta,ab OR 'mammary artery':ta,ab OR 'internal thoracic artery':ta,ab OR 'rima':ta,ab OR 'bima':ta,ab OR 'radial artery':ta,ab OR 'ra':ta,ab OR 'gastroepiploic artery':ta,ab OR 'rga':ta,ab 169,447

#3 #1 AND #2 6951

#4 'coronary artery bypass graft':ta,ab OR 'surgery':ab,ta OR 'cabg':ta,ab 1,714,538

#5 #3 AND #4 998

**Meta-analyses Of Observational Studies in Epidemiology Checklist**

| **Checklist Items** | | **Status** |
| --- | --- | --- |
| Reporting of background should include | | |
|  | Problem definition | COMPLETED |
|  | Hypothesis statement | DEFERRED |
|  | Description of study outcomes | COMPLETED |
|  | Type of exposure or intervention used | COMPLETED |
|  | Type of study designs used | COMPLETED |
|  | Study population | COMPLETED |
| Reporting of search strategy should include | | |
|  | Qualifications of searchers (eg, librarians and investigators) | COMPLETED |
|  | Search strategy, including time period included in the synthesis and keywords | COMPLETED |
|  | Effort to include all available studies, including contact with authors | COMPLETED |
|  | Databases and registries searched | COMPLETED |
|  | Search software used, name and version, including special features used (eg, explosion) | COMPLETED |
|  | Use of hand searching (eg, reference lists of obtained articles) | COMPLETED |
|  | List of citations located and those excluded, including justification | COMPLETED |
|  | Method of addressing articles published in languages other than English | DEFERRED |
|  | Method of handling abstracts and unpublished studies | DEFERRED |
|  | Description of any contact with authors | DEFERRED |
| Reporting of methods should include | | |
|  | Description of relevance or appropriateness of studies assembled for assessing the hypothesis to be tested | COMPLETED |
|  | Rationale for the selection and coding of data (eg, sound clinical principles or convenience) | COMPLETED |
|  | Documentation of how data were classified and coded (eg, multiple raters, blinding, and interrater reliability) | COMPLETED |
|  | Assessment of confounding (eg, comparability of cases and controls in studies where appropriate) | COMPLETED |
|  | Assessment of study quality, including blinding of quality assessors; stratification or regression on possible predictors of study results | COMPLETED |
|  | Assessment of heterogeneity | COMPLETED |
|  | Description of statistical methods (eg, complete description of fixed or random effects models, justification of whether the chosen models account for predictors of study results, dose-response models, or cumulative meta-analysis) in sufficient detail to be replicated | COMPLETED |
|  | Provision of appropriate tables and graphics | COMPLETED |
| Reporting of results should include | | |
|  | Graphic summarizing individual study estimates and overall estimate | COMPLETED |
|  | Table giving cescriptive information for each study included | COMPLETED |
|  | Results of sensitivity testing (eg, subgroup analysis) | COMPLETED |
|  | Indication of statistical uncertainty of findings | COMPLETED |
| Reporting of discussion should include | | |
|  | Quantitative assessment of bias (eg, publication bias) | COMPLETED |
|  | Justification for exclusion (eg, exclusion of non—English-language citations) | COMPLETED |
|  | Assessment of quality of included studies | COMPLETED |
| Reporting of conclusions should include | | |
|  | Consideration of alternative explanations for observed results | COMPLETED |
|  | Generalization of the conclusions (ie, appropriate for the cata presented and within the domain of the literature review) | COMPLETED |
|  | Guidelines for future research | COMPLETED |
|  | Disclosure of funding source | COMPLETED |


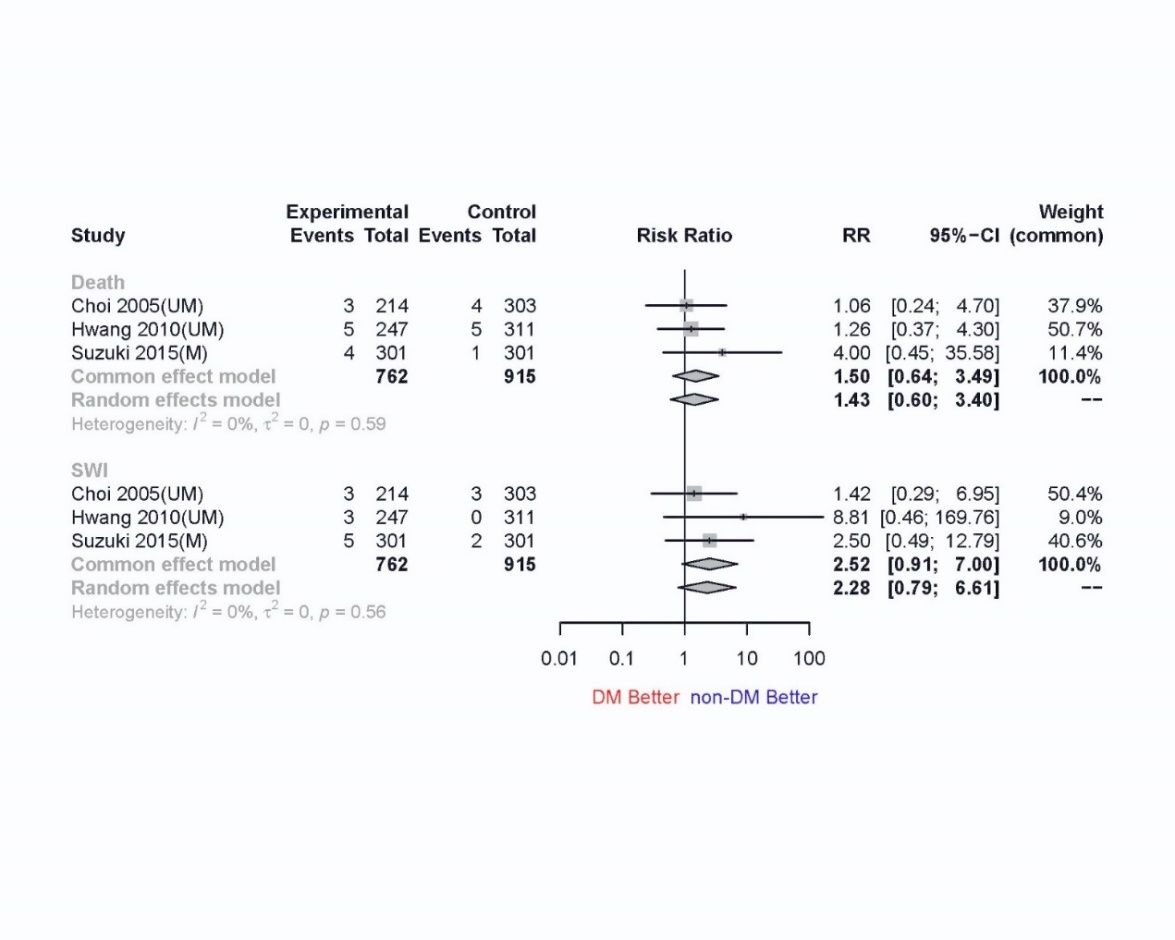


A


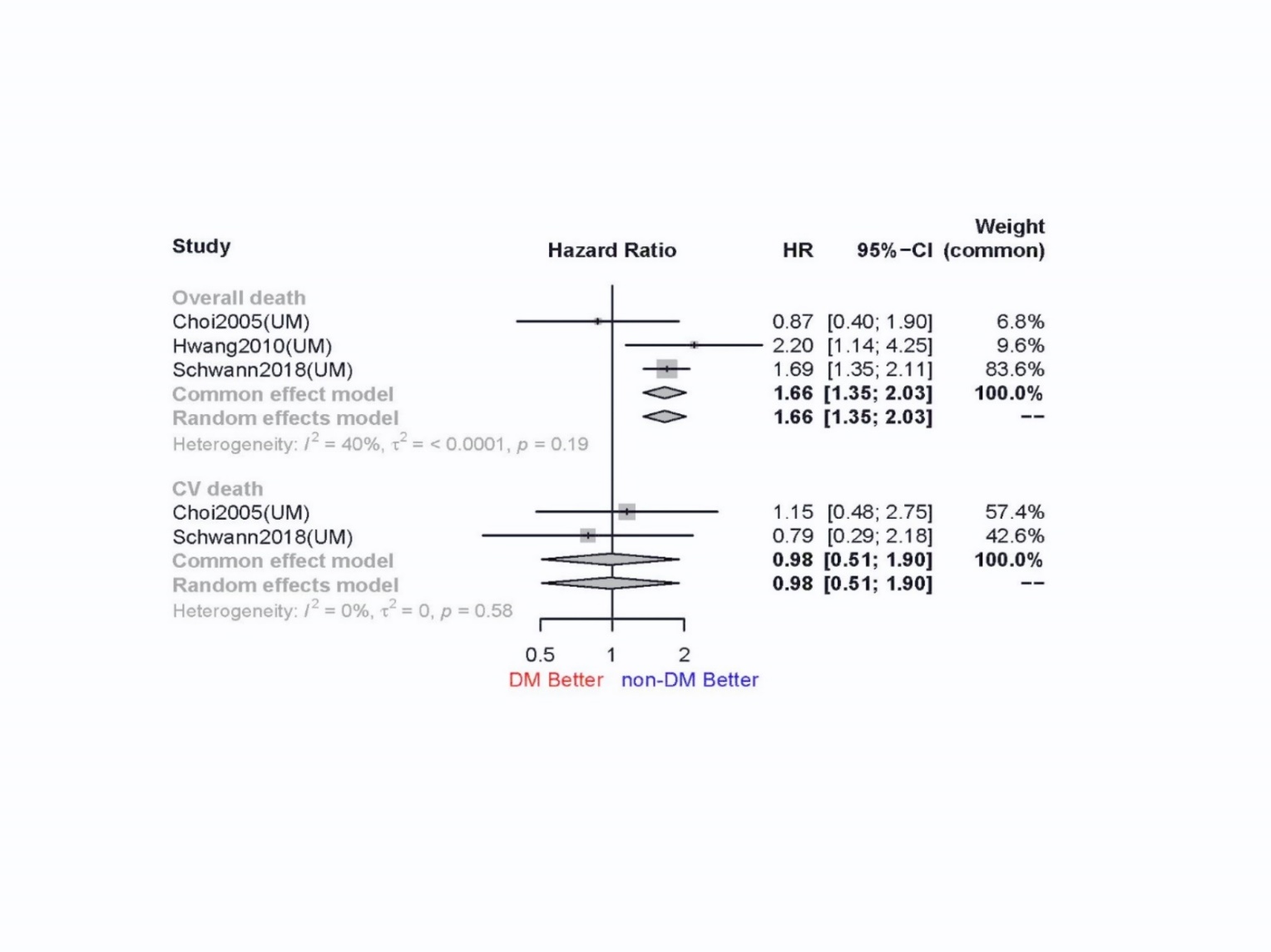


B

Figure S1. Plots for the clinical outcomes of TAR in diabetic and non-diabetic patients.

A). Forest plot of early death and any SWI. B) Forest plot of long-term death and cardiovascular death.

Note: M: the studies with data of matched cohorts; UM: the studies with data of unmatched cohorts; TAR: total arterial revascularization; CVR: conventional revascularization with veins; HR: hazard ratio; CI: confidence interval; DM: diabetes mellitus patients; non-DM: non-diabetes Mellitus patients.


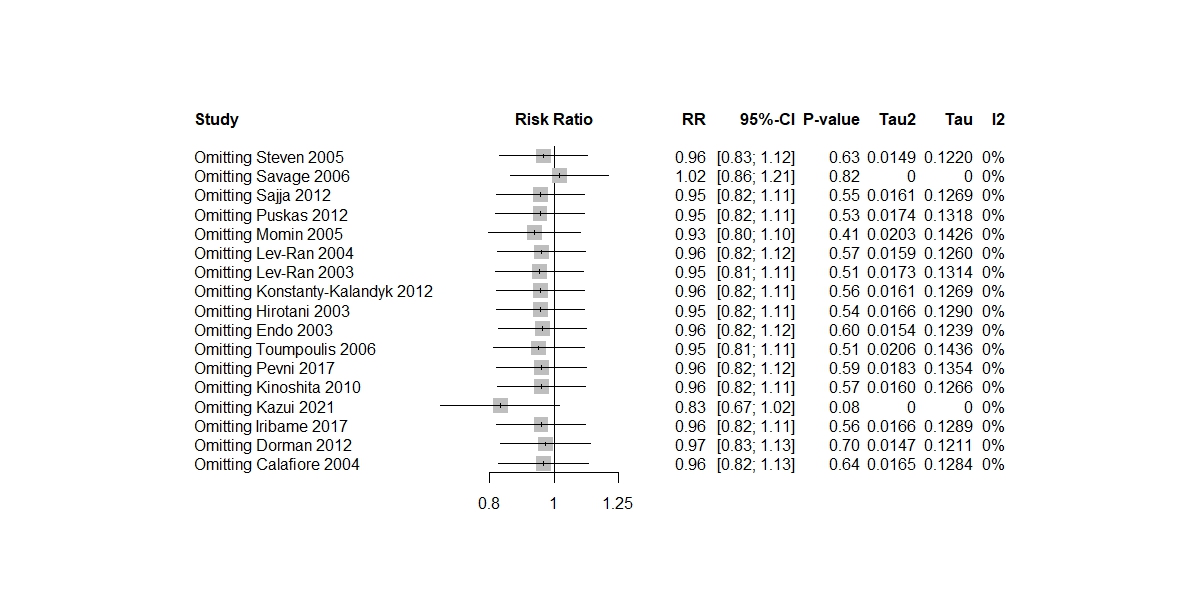


A


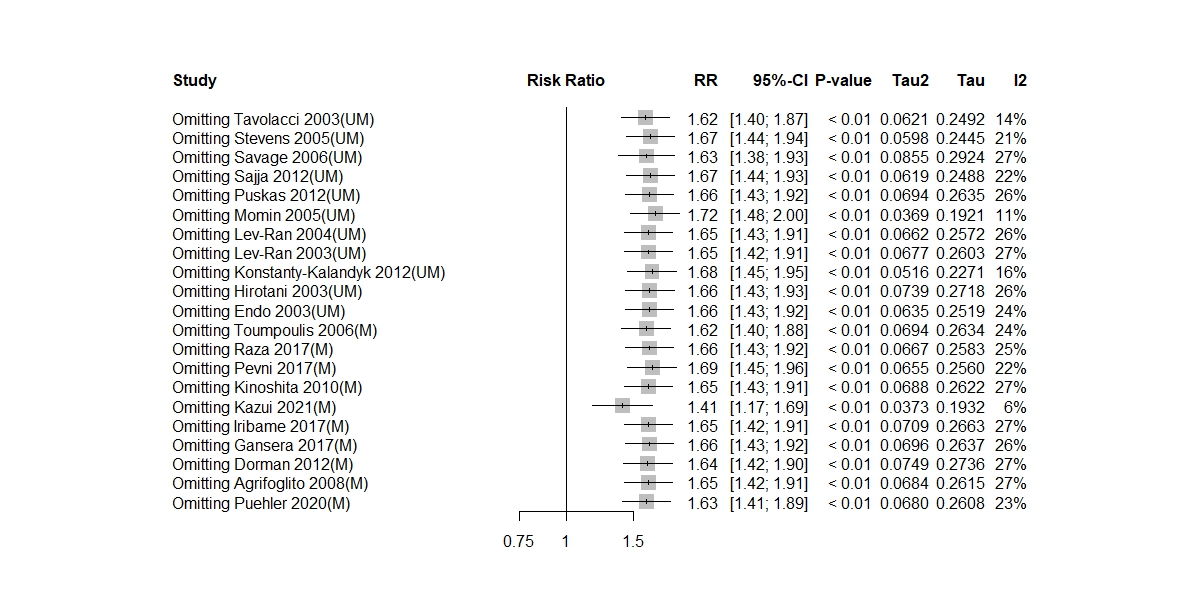


B


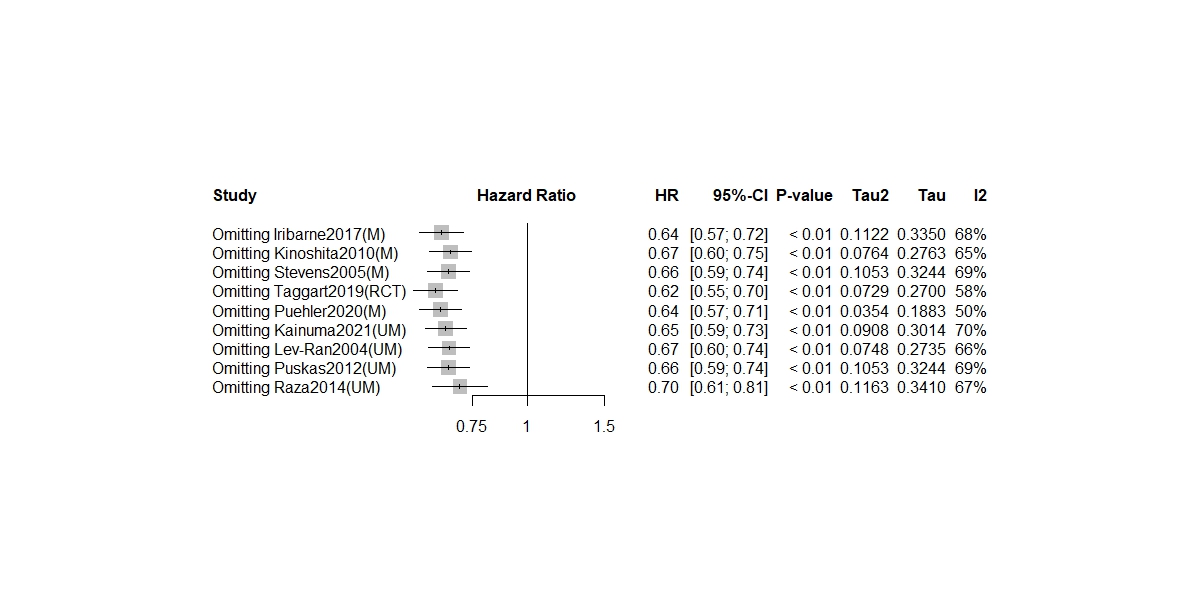


C


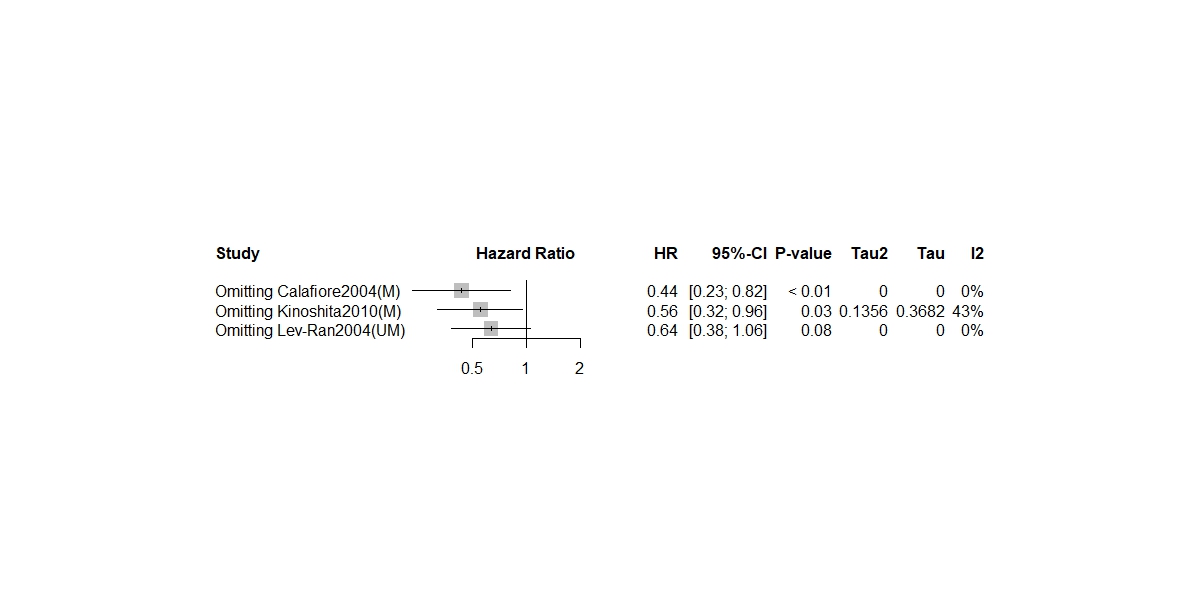


D

Figure S2. Sensitivity analyses of early death (A), SWI (B), long-term overall death (C) and cardiovascular death (D) in the comparison between BIMA and CVR in DM patients.


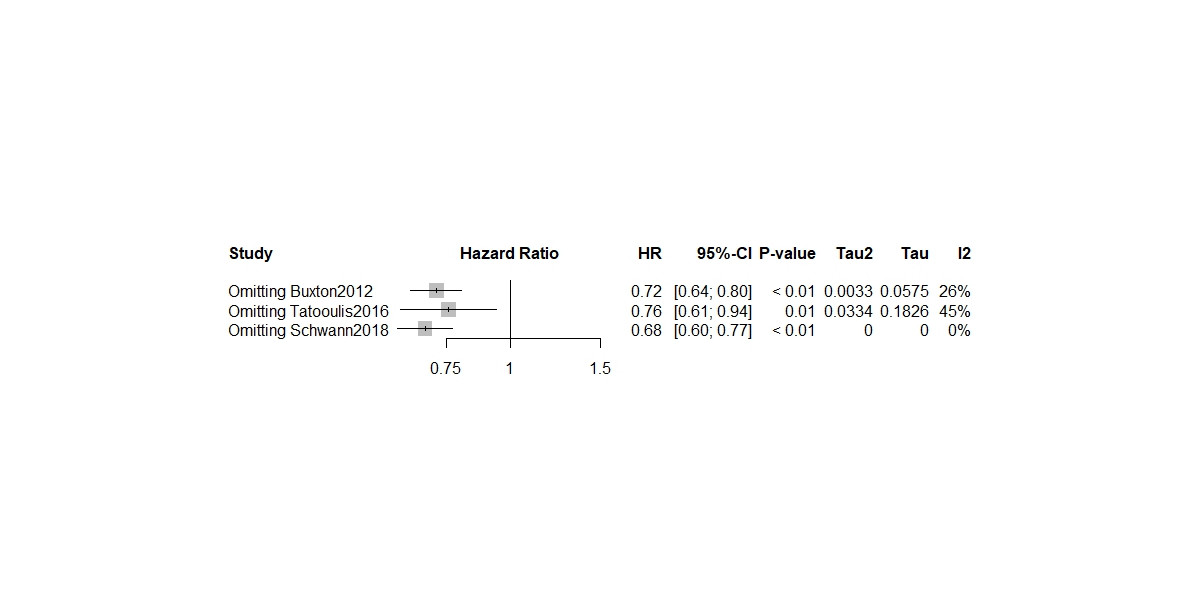


Figure S3. Sensitivity analyses of long-term overall death in the comparison between RA and CVR in DM patients.

| Study | Selection | | | | Comparability | Outcome | Follow-up | | Total score. |
| --- | --- | --- | --- | --- | --- | --- | --- | --- | --- |
|  | Representative of the exposed cohort. | Selection of the nonexposed cohort. | Ascertainment of exposure. | Demonstration that outcome of interest was not present at the start of the study. | Comparability of cohorts on the basis of the design or analysis. | Assessment of outcome. | Was follow-up long enough for outcomes to occur? | Adequacy of follow-up of cohorts. |  |
| Buxton2012 | ★ | ★ | ★ | ★ | ★★ | ★ | ★ | ☆ | 8 |
| DiBacco2019 | ★ | ★ | ★ | ★ | ★★ | ★ | ★ | ★ | 9 |
| Hwang2010 | ★ | ★ | ★ | ★ | ★☆ | ★ | ★ | ★ | 8 |
| Suzuki2005 | ★ | ★ | ★ | ★ | ★★ | ★ | ★ | ★ | 9 |
| Tatoulis2016 | ★ | ★ | ★ | ★ | ★☆ | ★ | ★ | ★ | 8 |
| Choi2005 | ★ | ★ | ★ | ★ | ★☆ | ★ | ★ | ☆ | 7 |
| Abelaira2021 | ★ | ★ | ★ | ★ | ★☆ | ★ | ★ | ☆ | 7 |
| Agrifoglio2008 | ★ | ★ | ★ | ★ | ★☆ | ★ | ★ | ☆ | 7 |
| Dorman2012 | ★ | ★ | ★ | ★ | ★★ | ★ | ★ | ☆ | 8 |
| Calafiore2004 | ★ | ★ | ★ | ★ | ★★ | ★ | ★ | ★ | 9 |
| Endo2003 | ★ | ★ | ★ | ★ | ★☆ | ★ | ★ | ★ | 8 |
| Gansera2017 | ★ | ★ | ★ | ★ | ★★ | ☆ | ★ | ★ | 7 |
| Hirotani2003 | ★ | ★ | ★ | ★ | ★☆ | ★ | ★ | ★ | 7 |
| Iribarne2017 | ★ | ★ | ★ | ★ | ★☆ | ★ | ★ | ★ | 8 |
| Kainuma2021 | ★ | ★ | ★ | ★ | ★★ | ☆ | ★ | ★ | 8 |
| Kazui2021 | ★ | ★ | ★ | ★ | ★★ | ☆ | ★ | ★ | 8 |
| Konstanty-Kalandyk2012 | ★ | ★ | ★ | ★ | ★☆ | ☆ | ☆ | ★ | 6 |
| Kinoshita2010 | ★ | ★ | ★ | ★ | ★☆ | ★ | ★ | ★ | 8 |
| Lev-Ran2004 | ★ | ★ | ★ | ★ | ★☆ | ★ | ★ | ★ | 8 |
| Lev-Ran2003 | ★ | ★ | ★ | ★ | ★☆ | ★ | ★ | ★ | 8 |
| Momin2005 | ★ | ★ | ★ | ★ | ★☆ | ★ | ☆ | ★ | 7 |
| Muneretto2006 | ★ | ★ | ★ | ★ | ★★ | ☆ | ★ | ☆ | 7 |
| Pevni2017 | ★ | ★ | ★ | ★ | ★★ | ★ | ★ | ☆ | 8 |
| Puskas2012 | ★ | ★ | ★ | ★ | ★☆ | ★ | ★ | ★ | 8 |
| Raza2017 | ★ | ★ | ★ | ★ | ★★ | ★ | ★ | ☆ | 8 |
| Raza2014 | ★ | ★ | ★ | ★ | ★☆ | ★ | ★ | ★ | 8 |
| Sajja2012 | ★ | ★ | ★ | ★ | ★☆ | ☆ | ☆ | ★ | 6 |
| Savage2006 | ★ | ★ | ★ | ★ | ★☆ | ☆ | ★ | ☆ | 6 |
| Stevens2005 | ★ | ★ | ★ | ★ | ★★ | ★ | ★ | ★ | 9 |
| Tavolacci2003 | ★ | ★ | ★ | ★ | ★☆ | ★ | ☆ | ★ | 7 |
| Toumpoulis2006 | ★ | ★ | ★ | ★ | ★☆ | ★ | ★ | ★ | 8 |
| Hoffman 2014 | ★ | ★ | ★ | ★ | ★★ | ★ | ★ | ☆ | 8 |
| Puehler2020 | ★ | ★ | ★ | ★ | ★★ | ★ | ★ | ☆ | 8 |

| **Study** | **D1** | **D2** | **D3** | **D4** | **D5** | **Overall** |
| --- | --- | --- | --- | --- | --- | --- |
| **Taggart2019** | **Low** | **Some concern** | **Low** | **Low** | **Low** | **Some concerns** |

Footnotes: D1: Risk of bias arising from the randomization process; D2: Risk of bias due to deviations from the intended interventions; D3: Risk of bias due to missing outcome data; D4: Risk of bias in the measurement of the outcome; D5: Risk of bias in the selection of the reported result; Overall: Overall risk of bias

Table 1. Results of quality assessment using the Newcastle-Ottawa Scale and Version 2 of the Cochrane tool for assessing risk of bias in randomized trial ( RoB2) for cohort study and randomized trial, respectively.
